# A one-year pilot study comparing direct-infusion high resolution mass spectrometry based untargeted metabolomics to targeted diagnostic screening for inherited metabolic diseases

**DOI:** 10.3389/fmolb.2023.1283083

**Published:** 2023-11-02

**Authors:** Anke P. Willems, Maria van der Ham, Birgit G. M. Schiebergen-Bronkhorst, Mirjam van Aalderen, Martina M. J. de Barse, Fini E. De Gruyter, Ilja N. van Hoek, Mia L. Pras-Raves, Monique G. M. de Sain-van der Velden, Hubertus C. M. T. Prinsen, Nanda M. Verhoeven-Duif, Judith J. M. Jans

**Affiliations:** Section Metabolic Diagnostics, Department of Genetics, University Medical Center Utrecht, Utrecht, Netherlands

**Keywords:** untargeted metabolomics, inherited metabolic diseases, direct-infusion high resolution mass spectrometry, biomarker, diagnostics, genetic diseases

## Abstract

**Background:** Early diagnosis of inherited metabolic diseases (IMDs) is important because treatment may lead to reduced mortality and improved prognosis. Due to their diversity, it is a challenge to diagnose IMDs in time, effecting an emerging need for a comprehensive test to acquire an overview of metabolite status. Untargeted metabolomics has proven its clinical potential in diagnosing IMDs, but is not yet widely used in genetic metabolic laboratories.

**Methods:** We assessed the potential role of plasma untargeted metabolomics in a clinical diagnostic setting by using direct infusion high resolution mass spectrometry (DI-HRMS) in parallel with traditional targeted metabolite assays. We compared quantitative data and qualitative performance of targeted versus untargeted metabolomics in patients suspected of an IMD (*n* = 793 samples) referred to our laboratory for 1 year. To compare results of both approaches, the untargeted data was limited to polar metabolites that were analyzed in targeted plasma assays. These include amino acid, (acyl)carnitine and creatine metabolites and are suitable for diagnosing IMDs across many of the disease groups described in the international classification of inherited metabolic disorders (ICIMD).

**Results:** For the majority of metabolites, the concentrations as measured in targeted assays correlated strongly with the semi quantitative Z-scores determined with DI-HRMS. For 64/793 patients, targeted assays showed an abnormal metabolite profile possibly indicative of an IMD. In 55 of these patients, similar aberrations were found with DI-HRMS. The remaining 9 patients showed only marginally increased or decreased metabolite concentrations that, in retrospect, were most likely to be clinically irrelevant. Illustrating its potential, DI-HRMS detected additional patients with aberrant metabolites that were indicative of an IMD not detected by targeted plasma analysis, such as purine and pyrimidine disorders and a carnitine synthesis disorder.

**Conclusion:** This one-year pilot study showed that DI-HRMS untargeted metabolomics can be used as a first-tier approach replacing targeted assays of amino acid, acylcarnitine and creatine metabolites with ample opportunities to expand. Using DI-HRMS untargeted metabolomics as a first-tier will open up possibilities to look for new biomarkers.

## 1 Introduction

Inherited metabolic diseases (IMDs) are a phenotypically heterogenous group of genetic diseases, now comprising 1881 diseases (IEMbase, 05-08-2023). Early diagnosis of IMDs is important, because disease-specific treatment may lead to reduced mortality and improved prognosis (Saudubray et al., 2006; Hoytema van Konijnenburg et al., 2021) and because it enables genetic counseling of families. It is a challenge to diagnose this diverse spectrum of diseases in a timely manner.

Current practice for diagnosing IMDs in genetic metabolic laboratories includes a selection of targeted metabolite platforms in blood and/or urine samples, the choice of which depends on the clinical description of the patient. Examples of commonly used platforms are amino acid and acylcarnitine analysis in plasma and organic acid analysis in urine. Although this targeted approach provides reliable results with accurate quantification, it lacks the flexibility to easily look beyond its established, fixed set of metabolites. In addition, multiple assays, each with their own sample preparation, are time consuming and labor intensive and can lead to long turnaround times. Given the increasing number of known IMDs and the concurrently expanding number of reported biomarkers needed for diagnosis (IEMbase), there is an emerging need for a comprehensive test to acquire a complete view of metabolite status.

Untargeted metabolomics encompasses an unbiased approach to measure all small molecules in a certain sample, within the limitations of the method, such as solubility and ionizability of the molecules. In recent years, the attention for untargeted metabolomics in diagnosing IMDs has been increasing and its clinical potential has been proven by several research groups using different approaches such as DI-HRMS (Haijes et al., 2019a; Haijes et al., 2019b), LC-QTOF-MS (Coene et al., 2018; Steinbusch et al., 2021), LC-HRMS (Bonte et al., 2019) or a combination of GC and LC approaches in parallel (Miller et al., 2015; Almontashiri et al., 2020; Liu et al., 2021) While chromatography based methods can separate metabolites with identical masses, direct-infusion approaches are faster, technically uncomplicated, need a very small amount of material, are robust, have a great potential for high-throughput analysis and bypass the need to create an experimental library of metabolite masses and retention times. Untargeted metabolomics can integrate a large number of metabolites into a single assay, reducing the complexity of sample preparation, increasing the number of metabolites assessed and eventually improving turnaround time. Unlike the traditional targeted approaches, untargeted metabolomics enables new biomarker discovery, both in established (Broeks et al., 2019; Haijes et al., 2019c; Kennedy et al., 2019; Engelke et al., 2021; van Outersterp et al., 2021) and in newly discovered IMDs (Mochel et al., 2009; van Karnebeek et al., 2016; Rodan et al., 2018). Finding new markers for established IMDs may be of great interest in relation to disease severity and progression, as well as in assessing treatment response (Pillai et al., 2020; Schoen and Singh, 2022; Tallis et al., 2022). In addition, analysis of a broad spectrum of metabolites will facilitate a better understanding of disease pathology, a higher diagnostic yield, and the possibility to look into interactions between metabolic pathways (Jans et al., 2022). However, integrating a diverse spectrum of chemically different metabolites into one platform is analytically challenging.

We and others have previously shown the clinical potential of untargeted metabolomics in cohorts of patients with IMDs (Miller et al., 2015; Coene et al., 2018; Haijes et al., 2019a; Haijes et al., 2019b; Bonte et al., 2019; Almontashiri et al., 2020; Steinbusch et al., 2021). We now present the results of a one-year unbiased pilot study directly running untargeted metabolomics in parallel with traditional targeted metabolic assays, for all patients suspected of an IMD referred to our laboratory. We compared quantitative data and qualitative performances of the two approaches and focus on metabolites in the amino acid, acylcarnitine and creatine metabolism, covering biomarkers for many of the disease groups described in the international classification of inherited metabolic disorders [ICIMD, (Ferreira et al., 2021)].

## 2 Materials and methods

### 2.1 Patient inclusion

**FIGURE 1:**
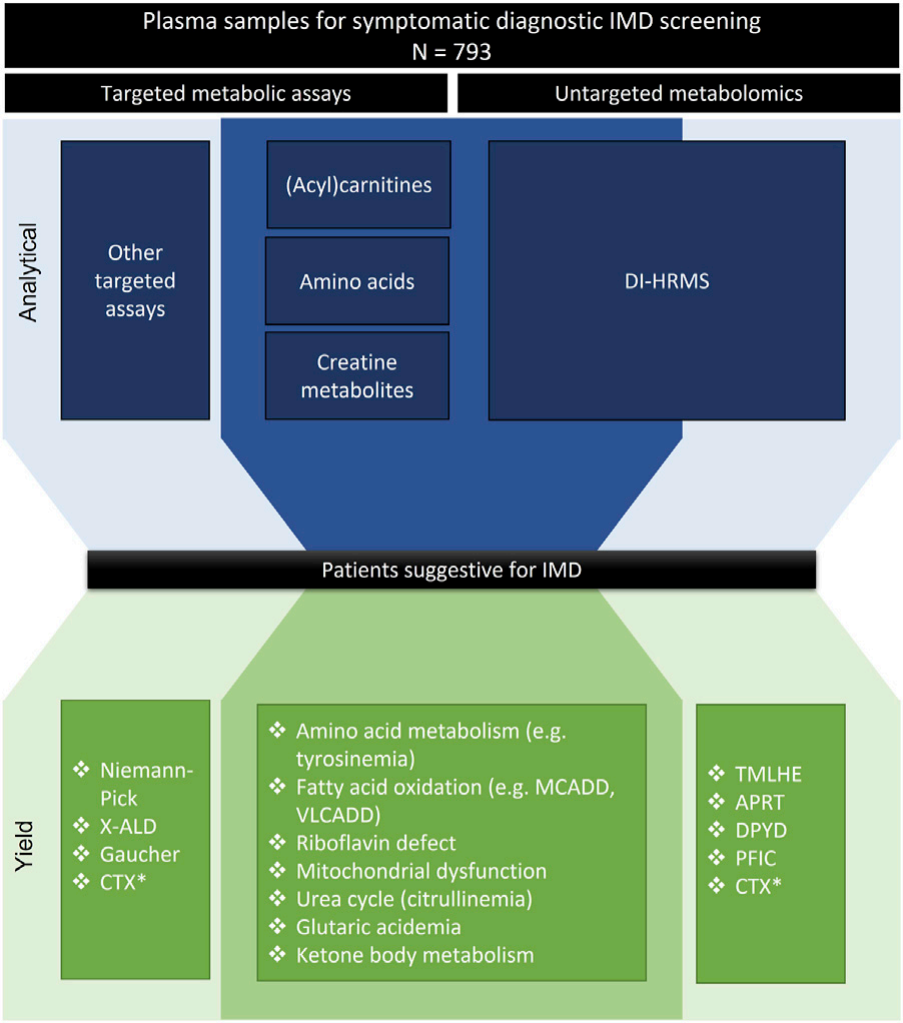
Overview of pilot study. Schematic view of pilot study comparing DI-HRMS untargeted metabolomics to targeted metabolic assays. The middle part (darker shade) represents assays and yield included in the pilot. The outer part (lighter shade) represents additional assays and yield. The lower part gives an overview of suspected diagnosis based on metabolite analysis, these are not (all) patients with confirmed diagnosis. *CTX is detected by both targeted and untargeted approaches, but based on different metabolites, therefore depicted here twice.

Heparinized plasma samples of patients referred to our genetic metabolic laboratory for symptomatic diagnostic screening of IMDs during a period of 1 year (2021) were included (*n* = 793). Samples received for disease monitoring, treatment follow-up or for confirmation of a specific disease, e.g., based on a newborn screening referral or for biochemical validation of a reported gene mutation, were excluded. Traditional targeted metabolic screening and untargeted metabolomics were always performed in parallel using plasma from the same vial for each sample (overview of pilot study in Figure 1).

### 2.2 Targeted metabolic analyses

Based on the symptoms of the patient, provided by the requesting physicians, a panel of targeted metabolite analyses was performed. The assays performed in this cohort were validated according to ISO standards and included assays for amino acids, acylcarnitines, methylmalonic acid and homocysteine, intermediates of cholesterol biosynthesis, oxysterols and very long-chain fatty acids. Analyses of creatine, guanidinoacetate and succinyl acetone were only performed in specific cases. Only amino acid, acylcarnitine, creatine and guanidinoacetate data were used for comparison with untargeted metabolomics.

Briefly, amino acids were quantified without derivatization using HILIC (hydrophilic interaction liquid chromatography) MS/MS (tandem mass spectrometry) as described in (Prinsen et al., 2016). Carnitine and acylcarnitines were analyzed by flow injection UHPLC (ultra-high performance liquid chromatography) MS/MS. MRM (multiple reaction monitoring) transitions of butyl ester derivatives of acylcarnitines and internal standards were analyzed in positive electro spray ionization (de Sain-van der Velden et al., 2013). Creatine and guanidinoacetate were analyzed using UHPLC-MS/MS with column separation. The typical turnaround time for one targeted assay is 1–2 days, the turnaround time for the total of metabolite assays for one patient is typically 2 weeks.

### 2.3 Untargeted metabolomics by DI-HRMS

Untargeted metabolomics analysis including sample preparation and data analysis was performed as described in (Haijes et al., 2019b). Samples were analyzed in 46 technical runs, resulting in an almost weekly analysis. Control samples in each run consisted of 30 randomly selected samples from a batch of 60 anonymized samples of patients in whom an IMD was excluded after performance of a thorough diagnostic metabolic screening. The batch of control samples was composed of patients varying in age and sex. Hemolytic plasma samples and samples from patients with specific diets (e.g., ketogenic diet) were not included as control. Three IMD positive controls (patients with lysinuric protein intolerance, phenylketonuria and propionic acidemia) were included, chosen because of diagnostic metabolites with different chemical properties and covering analysis in both positive and negative ion mode. Semi-quantitative data for each metabolite in each patient was obtained by calculating Z-scores (number of standard deviations from controls) as described in (Haijes et al., 2019b). The total turnaround time for one sample batch was approximately 2 days.

### 2.4 Metabolite selection

To be able to compare results from both approaches, we limited the untargeted data to polar metabolites that were also measured in targeted plasma analysis: amino acids, (acyl)carnitines, creatine and guanidinoacetate. Succinyl acetone was not included because of the low number of data points. Homocysteine was not included because targeted plasma homocysteine analysis involves DTT treatment leading to quantification of total homocysteine while methanol-based sample extraction as performed in DI-HRMS only captures the unbound fraction of homocysteine resulting in incomparable data (Haijes et al., 2019b). Although we have previously shown that our method correctly captures and annotates methylmalonic acid, enabling identification of patients with methylmalonic aciduria (Haijes et al., 2019b), we chose to exclude it for this study since Z-scores obtained by DI-HRMS correlate poorly in the lower concentration range (Supplementary Figure SF1) due to presence of isomeric metabolites (e.g., succinic acid) in high concentrations. This untargeted metabolomics approach including sample preparation is not suitable for large lipophilic metabolites such as very long chain fatty acids, oxysterols and cholesterol intermediates.

### 2.5 Statistical analyses

Z-scores of the selected metabolites were compared to concentrations measured by quantitative, targeted analyses performed as part of the traditional metabolic screening. The correlation between Z-scores and quantitatively derived concentrations was assessed using Pearson’s correlation coefficient.

## 3 Results

### 3.1 Description of pilot study

#### 3.1.1 Cohort characteristics

**FIGURE 2:**
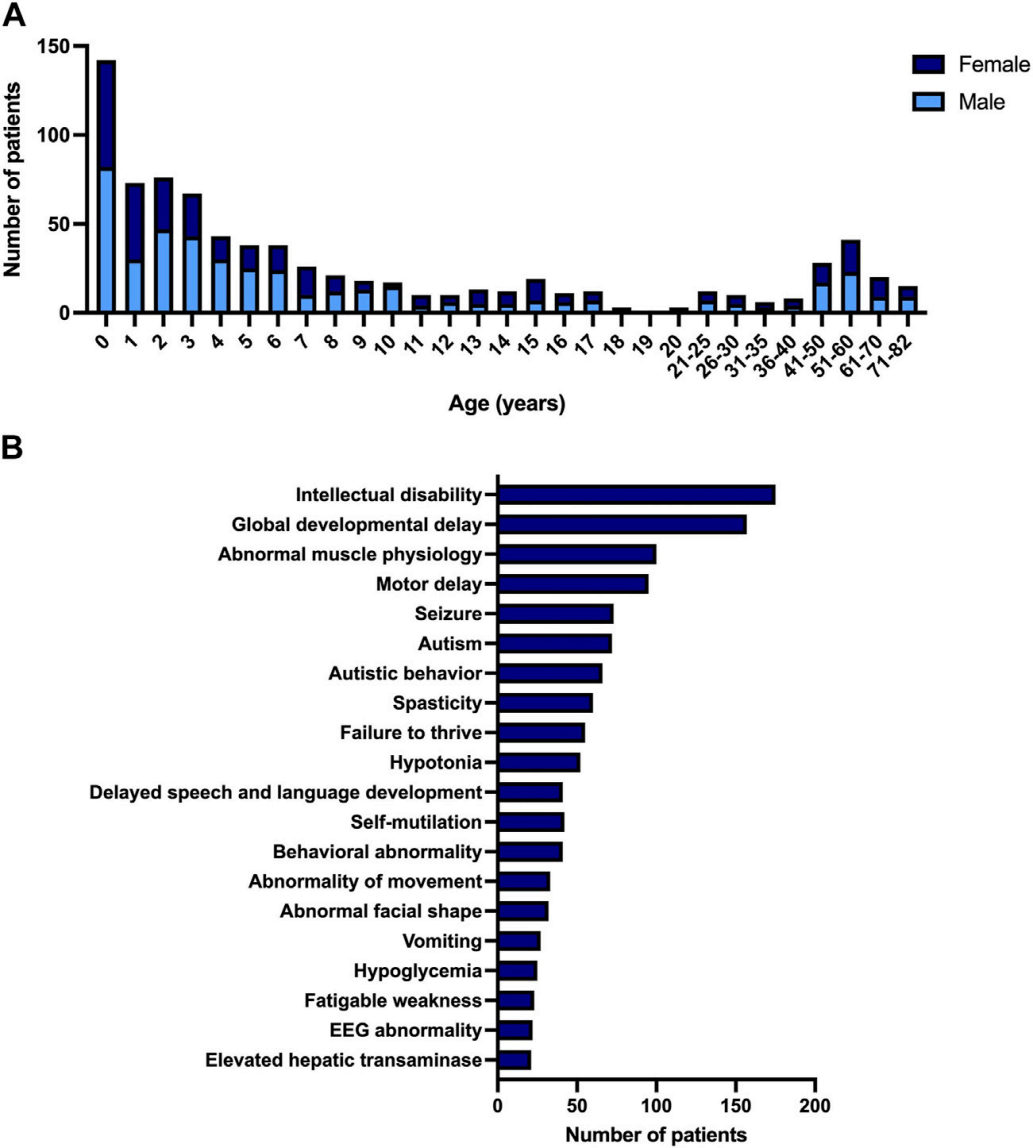
Description of pilot study. **(A)** Age and gender of patient population referred for symptomatic diagnostic screening of IMDs. **(B)** Most observed phenotypes in patient population referred for symptomatic diagnostic screening of IMDs, according to HPO systematic.

During the period of 1 year, our genetic metabolic laboratory received 793 plasma (or blood) samples from unique patients referred for symptomatic diagnostic screening of IMDs. These patients have a median age of 4 years and 10 months, ranging from 0 to 82 years old. The cohort is skewed towards male individuals (57% male). Most of the patients are children; 81% were younger than 18 years and 18% were younger than 1 year old (Figure 2A).

#### 3.1.2 Phenotypes

The phenotypic variability observed in IMDs is reflected by the number of clinical symptoms that are present in the cohort. We converted the symptoms reported by the referring physicians into corresponding HPO (Human Phenotype Ontology, (Kohler et al., 2021)) terms. In total 327 unique HPO terms were used. The most frequent HPO terms were intellectual disability and developmental delay, present in 175 and 157 patients respectively. Most of the patients with these symptoms were children (96%). The third most frequently seen clinical symptom was abnormal muscle physiology, mostly seen in adults (54%). Figure 2B gives an overview of the frequency of the most observed HPO terms (top 20).

#### 3.1.3 Targeted metabolic analyses

The most frequently performed combination of targeted analyses in our cohort are the assays of plasma amino acids, acylcarnitines and methylmalonic acid and homocysteine. This panel of analyses is performed in all children with intellectual disability and/or development delay, as recommended in the guideline of the Dutch pediatric association (NVK, 2018), and therefore reflects the large percentage of children with these clinical symptoms in our cohort. In a relatively large proportion of patients (7%), the targeted metabolic screening was limited to only the analysis of very long-chain fatty acids. This group consists of adult patients with spasticity in whom X-ALD (X-linked adrenoleukodystrophy) is investigated as a treatable cause of adult-onset hereditary spastic paraplegia. An overview of the frequencies of metabolic analyses performed in the cohort is displayed in Supplementary Figure SF2. In addition to analysis of metabolites (but not part of the scope of this paper) we performed screening for congenital disorders of glycosylation by transferrin isoelectric focusing in most of the plasma samples and chitotriosidase activity as a general lysosomal marker based on specific symptoms. Also, additional metabolic tests in urine and/or dried blood spot were performed in most patients.

### 3.2 Quantitative performance

Pearson correlation analysis shows a significant (*p* < 0.05) positive correlation between plasma concentrations measured with targeted metabolic assays and Z-scores determined with untargeted metabolomics for all amino acids, most acylcarnitines, creatine and guanidinoacetate. Exceptions are C4-DC-carnitine, C5-OH-carnitine, C12-DC-carnitine, C14-OH-carnitine, C16-OH-carnitine and C18-OH-carnitine, these metabolites show no (significant) positive correlation (Supplementary Figures SF3–SF5; Supplementary Tables ST1–ST3). Due to the DI approach without chromatography, isomeric metabolites such as leucine and isoleucine cannot be separated. However, the semi-quantitative Z-score for the m/z corresponding to leucine/isoleucine correlated well with both the quantitative concentration of leucine, as well as of isoleucine (Supplementary Figure SF3). Supplementary Table ST4 includes all intensity and Z-score data as detected by DI-HRMS that were used for the comparisons.

### 3.3 Qualitative performance

#### 3.3.1 Samples with abnormal metabolites

**FIGURE 3:**
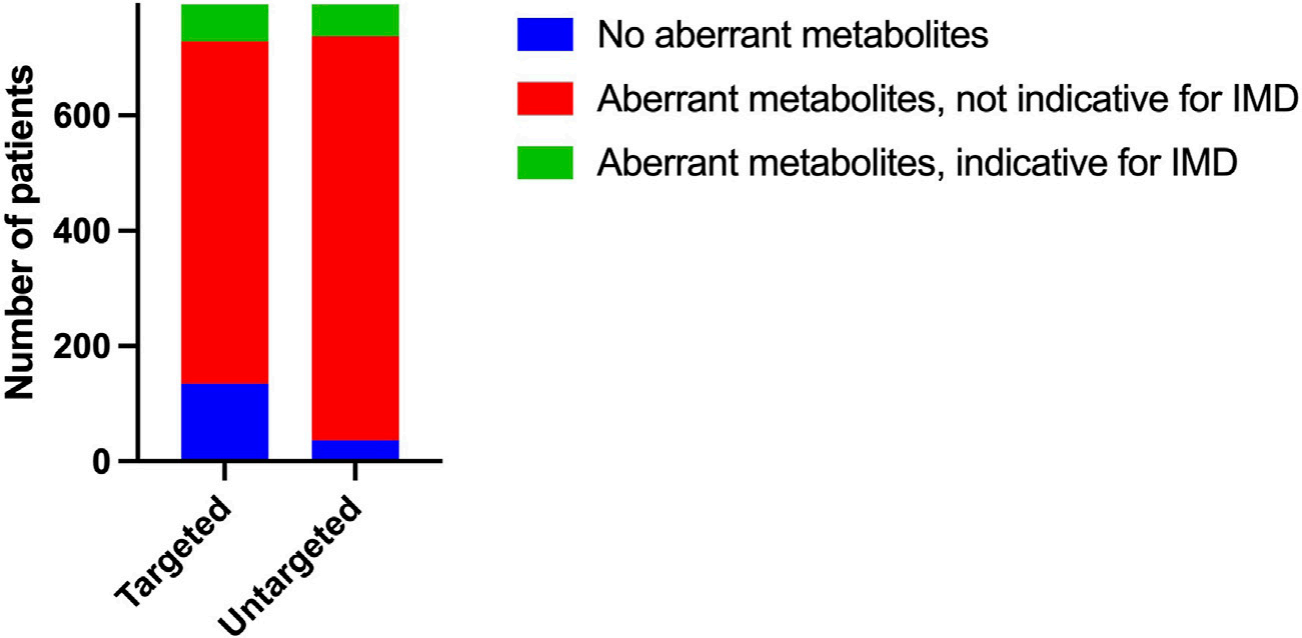
Qualitative performance Number of plasma samples with aberrant (amino acid, acylcarnitine and creatine) metabolites based on reference range for targeted metabolic assays and based on Z-score limits *Z* = 2 and *Z* = −1,5 for untargeted metabolomics. Number of plasma samples that showed aberrant (amino acid, acylcarnitine and creatine) metabolites possibly indicative of an IMD.

The result of a targeted metabolic diagnostic screening has traditionally been composed of quantitative data compared to reference ranges and an interpretation of these data in relation to the patients’ clinical symptoms by a laboratory specialist. The traditional targeted assays revealed aberrant metabolites in 83% (658/793) of plasma samples (Figure 3). This includes all metabolites (in amino acid, carnitine and creatine metabolism) elevated or decreased when strictly compared to the reference range used in patient diagnostics in our laboratory. These reference ranges are either based on literature or have been determined in-house, depending on the analysis; some of them are age-dependent. Z-scores calculated from untargeted metabolomics data showed at least one aberrant metabolite in 95% (756/793) of plasma samples (Figure 3). Upper and lower limits were set at *Z* = 2 and *Z* = −1,5 respectively for increased and decreased Z-scores. Upper and lower limits were used for amino acids, carnitine, creatine and guanidinoacetate. For acylcarnitines, we used only upper limits.

#### 3.3.2 Samples with possible indications for IMDs

To assess whether our untargeted approach would detect all aberrations that are relevant to diagnosing IMDs, we selected all patients with metabolite aberrations that may be indicative of IMDs reported on targeted metabolic screening. In 8% (64/793) of the samples, targeted metabolic assays revealed metabolite aberrations that could be an indication of an IMD.

This group includes patients with very clear clues for a specific IMD (e.g., urea cycle defect, glutaric aciduria, defect in ketone body metabolism, tyrosinaemia) and patients with aberrations that could be well explained by an IMD but could also be caused by exogenous factors (mainly acylcarnitines indicative of fatty acid oxidation defects or riboflavin defects). In addition, it includes a large group of patients with non-specific metabolites that could possibly be explained by mitochondrial disorders or secondary mitochondrial dysfunction, mainly elevated alanine and proline. Supplementary Table ST5 shows an overview of these samples. Most of the aberrations found in targeted assays were also reflected in data from the untargeted approach. In 9 plasma samples, untargeted metabolomics did not detect potentially relevant aberrant metabolite levels that were reported in targeted metabolic assays, possibly because these aberrations were very mild. These aberrations included a slightly elevated C18:1-carnitine and mild aberrations of proline, alanine and citrulline in the context of possible mitochondrial dysfunctions.

### 3.4 Other indications for IMDs

Our cohort included patients’ samples that in the untargeted approach showed potentially relevant abnormal levels of metabolites that were not included in our targeted plasma assays (Figure 1). For example, our cohort included four patients with abnormal concentrations of purine or pyrimidine metabolites in urine, which were also clearly detected by DI-HRMS in plasma. Also, the cohort included two patients with high levels of plasma bile acids that were detected by DI-HRMS. Analysis of total plasma bile acids is traditionally performed by routine clinical laboratories and therefore not part of specific IMD screening. In addition, we found a patient with a suspected ε-N-trimethyllysine hydroxylase (TMLHE) defect by DI-HRMS, that could not be detected by our targeted plasma screening. An abnormal bile acid profile in a patient suspected to have CTX (Cerebrotendinous Xanthomatosis) was detected both by targeted bile acid screening in urine and DI-HRMS in plasma. Our cohort included also patients’ samples with potentially relevant abnormalities that could not be detected by DI-HRMS (Figure 1), such as abnormal lipophilic metabolites that were indicative of Niemann-Pick B disease (oxysterols) and X-ALD (very long chain fatty acids). As explained, these metabolites are not compatible with our DI-HRMS assay due to the sample preparation. Lastly, the cohort included one patient with Gaucher disease that was not detected based on metabolite assays but based on enzymatic screening (abnormal plasma chitotriosidase and dried blood spot glucocerebrosidase activity).

## 4 Discussion

In this one-year pilot study we have run DI-HRMS untargeted metabolomics in parallel with traditional targeted metabolite assays in plasma of patients suspected of an IMD. Unlike previous studies mainly focusing on cohorts with patients already diagnosed with an IMD (Miller et al., 2015; Coene et al., 2018; Haijes et al., 2019a; Haijes et al., 2019b; Bonte et al., 2019; Almontashiri et al., 2020; Steinbusch et al., 2021), we aimed to compare data from both approaches in a representative cohort in daily metabolic laboratory practice, aspiring partial replacement of targeted plasma metabolite assays by initial screening by DI-HRMS.

One of the well-recognized advantages of untargeted metabolomics compared to targeted metabolite assays is reduction of time and labor. The turn-around-time of DI-HRMS untargeted metabolomics for one batch of samples in this pilot was typically 2 days, including sample preparation, runtime, data analysis and interpretation. While some individual targeted metabolite assays can be faster, running several targeted assays in parallel with one untargeted platform promptly shows the benefits of the latter in terms of number of technicians, hands-on time and amount of equipment needed. In some emergency situations, targeted assays as a first-tier may remain the method of choice, while in other emergency situations untargeted metabolomics may result in faster diagnosis because the simultaneous analysis of multiple metabolites is required. From this pilot study we can conclude that DI-HRMS untargeted metabolomics application as part of a first screen for diagnosing IMDs is practically feasible and does not delay metabolic screening. In the future, expanding the number of included metabolites in DI-HRMS untargeted metabolomics will reduce the number of targeted analyses that need to be performed in parallel, thereby reducing total turnaround time and labor per patient.

One of the limitations of most untargeted metabolomics approaches, including ours, is that it generates semi-quantitative data. Since a potentially very large number of metabolites can be analyzed, it is not feasible to include internal standards for all metabolites of interest. Also, highly abundant ions suppress the signal of ions with lower abundance, so the intensity measured for one metabolite is dependent on the presence of other metabolites. Taking these limitations in account we aimed to assess the correlation between quantitative concentration analyzed by targeted assays versus semi-quantitative Z-score analyzed by DI-HRMS for amino acid, acylcarnitine and creatine metabolites. We used Z-scores as a read-out for semi-quantitative intensity data to be able to include data from different analytic runs.

For most metabolites, semi-quantitative Z-scores obtained by untargeted metabolomics correlated well with concentrations analyzed by targeted platforms. For some metabolites (C4-DC-carnitine, C5-OH-carnitine, C12-DC-carnitine, C14-OH-carnitine, C16-OH-carnitine and C18-OH-carnitine) no or a less pronounced correlation was found. This may have various reasons. First, these metabolites are present in very low concentrations in plasma, close to the limit of detection. This results in the exclusion of several samples for quantitative comparison of these metabolites, because they were not detected (Supplementary Table ST2). In addition to the lower number of data points, this also leads to data that is grouped in a small number of distinct outcomes, instead of a continuous range of concentrations (C12-DC-carnitine and C18-OH-carnitine represent extreme examples, but the phenomenon can be seen in all acylcarnitines with a concentration range roughly below 0,1 μM, Supplementary Figure SF4). Also, unknown ions with identical m/z ratio that are present in plasma samples in higher abundance than these low abundant acylcarnitines may results in erroneously high Z-scores. This effect may be larger than in some other untargeted metabolomic approaches, because due to the direct infusion approach metabolites cannot be separated based on retention time. Secondly, the variation in acylcarnitine concentrations within the cohort is limited. For most acylcarnitines all data points are in the normal or only mildly elevated range (Supplementary Figure SF4). Addition of patients with clinically relevant elevated concentrations of these metabolites will increase correlation. For example, in samples from patients with 3-methylcrotonylglycinuria Z-scores for C5-OH-carnitine were up to 478 and in samples from patients with methylmalonic aciduria Z-scores for C4-DC-carnitine were up to 40 (data not shown, these patients were not is this cohort). In short, we conclude that quantitative concentrations of amino acid, acylcarnitine and creatine metabolites measured by targeted assays correlate well with semi-quantitative Z-scores obtained by DI-HRMS. Some acylcarnitines that are normally present in very low concentrations in plasma do not correlate well and may show false positives, illustrating the necessity to confirm untargeted data by quantitative assays.

To assess whether DI-HRMS untargeted metabolomics can be used as a first-tier approach instead of targeted assays of amino acids, acylcarnitines and creatine metabolites we investigated if samples showed metabolite abnormalities, both objectively (when compared to reference ranges) and by expert interpretation.

The targeted assays, in which metabolite concentrations are compared to reference ranges, revealed a lower percentage of plasma samples with abnormalities than the untargeted assay in which semi-quantitative Z-scores were used. This phenomenon can be easily explained. First, the effect of multiple testing is larger in the untargeted data than in quantitative targeted data, since we analyzed all selected metabolites in all patients by untargeted metabolomics while targeted assays were only performed if clinical phenotypes or other indications gave rise to it. Second, the limits of −1,5 to 2 will statistically generate more aberrant metabolites than the use of a reference range, which is mostly the mean ± 2SD, assuming that the data have a normal distribution. Third, the number of samples exceeding the reference range is strongly dependent on the size of the population in which a reference range is established. For targeted analyses, this population is usually larger (and thus more diverse) than the 30 control samples that are included in each untargeted metabolomics run.

We conclude that untargeted metabolomics with Z-score limits of *Z* = 2 and *Z* = −1,5 results in a large percentage of samples with aberrant metabolites. Future experience will reveal whether adjusted Z-score limits per metabolite can reduce the number of positive samples, without missing IMD diagnoses. However, because metabolite levels are subject to external factors such as age, nutrition, medication, and health status, a wide variation can be observed and deviation from the reference range is not always significant in itself. Therefore, like for quantitative data, an expert interpretation (and/or a diagnostic algorithm) will remain necessary for translation of semi-quantitative results to a meaningful conclusion.

Based on this expert opinion, we selected all patients with abnormal concentrations of amino acid, acylcarnitine and creatine metabolites in plasma that could point to an IMD and compared the concentration of these metabolites to the semi-quantitative Z-score obtained by DI-HRMS. All aberrant metabolites that specifically and clearly indicated an IMD diagnosis were also detected with DI-HRMS (Supplementary Table ST5). Abnormal metabolites that could be well explained by an IMD but could also be caused by exogenous factors were also detected with DI-HRMS, with the exception of a single mildly elevated C18:1-carnitine. Although theoretically this could be indicative for CPT2 (Carnitine palmitoyltransferase 2) deficiency, this is unlikely for this patient because of the mild elevation and the normal concentration of other acylcarnitines (de Sain-van der Velden et al., 2013). A large part of the IMD-related abnormal metabolites were non-specific metabolites that could be explained by mitochondrial disorders or secondary mitochondrial dysfunction. The elevated mitochondrial markers were almost all detected with DI-HRMS. However, in 7 patients mild elevations of alanine and/or proline and in 1 patient mildly decreased citrulline were not found. To the best of our knowledge, none of these patients has been diagnosed with a mitochondrial disorder to date. These type of abnormalities are observed frequently and are often subtle, non-specific and normalize over time, generally not leading to an IMD diagnosis. Within the limitations of our pilot study it is impossible to assess to which extent these mild amino acid aberrations are clinically relevant and therefore we cannot determine whether replacing targeted amino acid analysis by untargeted metabolomics would lead to missed patients with mitochondrial dysfunction or to reduced ‘false positives’ (Forny et al., 2021).

We conclude that DI-HRMS untargeted metabolomics can be used as a first-tier approach instead of targeted assays of amino acids, acylcarnitines and creatine biosynthesis metabolites without the risk of missing clear IMD diagnoses. Confirmation of diagnoses by targeted analysis will remain needed because the semi-quantitative nature of untargeted metabolomics may lead to false positives.

For the purpose of comparison, we limited all (including untargeted) data in this report to amino acid, acylcarnitine and creatine metabolites. However, additional (not confirmed) diagnoses in the cohort already illustrate the possibilities of expanding beyond these metabolite groups. We found 4 patients with purine or pyrimidine defects and 1 patient with CTX by abnormal purine/pyrimidine metabolites and bile acids in plasma, respectively, as analyzed by DI-HRMS. These metabolites are traditionally analyzed in targeted urine assays. The detection of high amounts of plasma bile acids by DI-HRMS also identified patients with PFIC1 and PFIC2, diseases that cannot be diagnosed by determining bile acid profiles in urine. In addition, we diagnosed a patient with ε-N-trimethyllysine hydroxylase (TMLHE) defect by DI-HRMS, that could not be detected by our targeted plasma screening.

On the other hand, DI-HRMS untargeted metabolomics will never completely replace targeted metabolic assays. One of the underlying reasons comprises the large variation in molecular structure of metabolites, illustrated by patients with Niemann-Pick B disease and X-ALD in our cohort. These patients were diagnosed based on elevated oxysterols and very long chain fatty acids, respectively; lipophilic metabolites that are not compatible with our sample work-up for the DI-HRMS platform. A different sample preparation approach, a specific lipidomics platform (Vaz et al., 2015) or a set of targeted assays for these metabolites will remain necessary to cover this part of the IMD spectrum. Additional methods may also remain necessary for other metabolites that require specific sample preparation (such as DTT treatment for total homocysteine) and for quantitative treatment monitoring in diagnosed patients, specifically for metabolites with endogenous or exogenous isomers that can be present in high amounts in human samples. Examples include separation of leucine and isoleucine in patients with amino acid disorders and analysis of moderately elevated methylmalonic acid such as in patients with cobalamin disorders or vitamin B12 deficiency. In addition, the semi-quantitative nature of DI-HRMS will always require confirmation with a targeted, quantitative assay.

In conclusion, a one-year pilot study showed that DI-HRMS untargeted metabolomics can be used as a first-tier approach replacing targeted assays of amino acid, acylcarnitine and creatine metabolites. Future studies will focus on expanding the number of validated diagnostic metabolites, thereby drastically reducing the number of patients for whom targeted assays of polar metabolites will be necessary. In addition, future research will focus on an integrated untargeted platform to analyze large lipophilic molecules, to further reduce time and labor. This future parallel untargeted approach opens up possibilities to easily look for new biomarkers across the entire IMD spectrum.

## Data Availability

The raw data supporting the conclusion of this article will be made available by the authors, without undue reservation.
