# Supplementary material for: A one-year pilot study comparing direct-infusion high resolution mass spectrometry based untargeted metabolomics to targeted diagnostic screening for inherited metabolic diseases
Source: Front Mol Biosci. 2023 Nov 2;10:1283083. doi: 10.3389/fmolb.2023.1283083 (PMC10657655; doi:10.3389/fmolb.2023.1283083)
Supplement: Supplementary file 1 [file DataSheet1.pdf]

Supplemental figures and tables

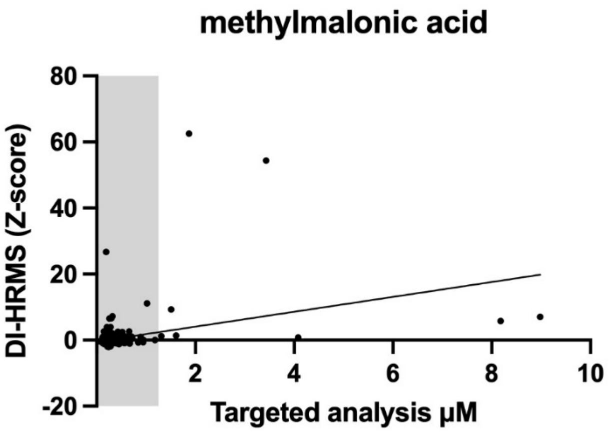

Supplemental figure SF1: methylmalonic acid correlation plot of quantitative concentration analyzed by targeted assays versus semi-quantitative Z-score analyzed by DI-HRMS.

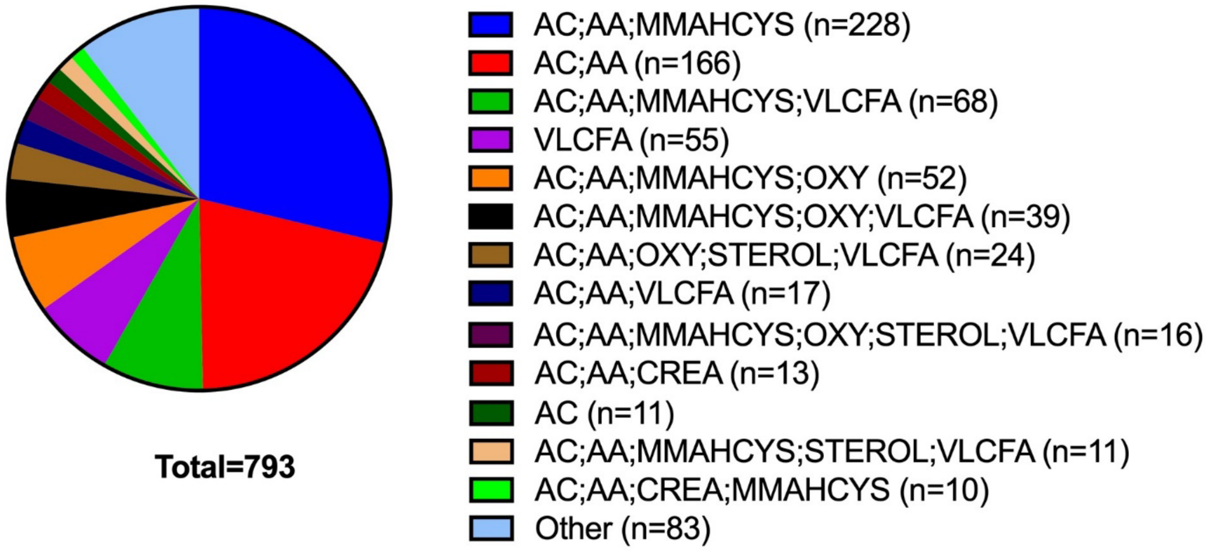

Supplemental figure SF2 Frequency of performed targeted metabolic analyses in patient cohort. AC: acylcarnitines, AA: amino acids, MMAHCYS: methylmalonic and homocysteine, VLCFA: very long chain fatty acids, OXY: oxysterols, STEROL: cholesterol intermediates, CREA: creatine and guanidinoacetate.

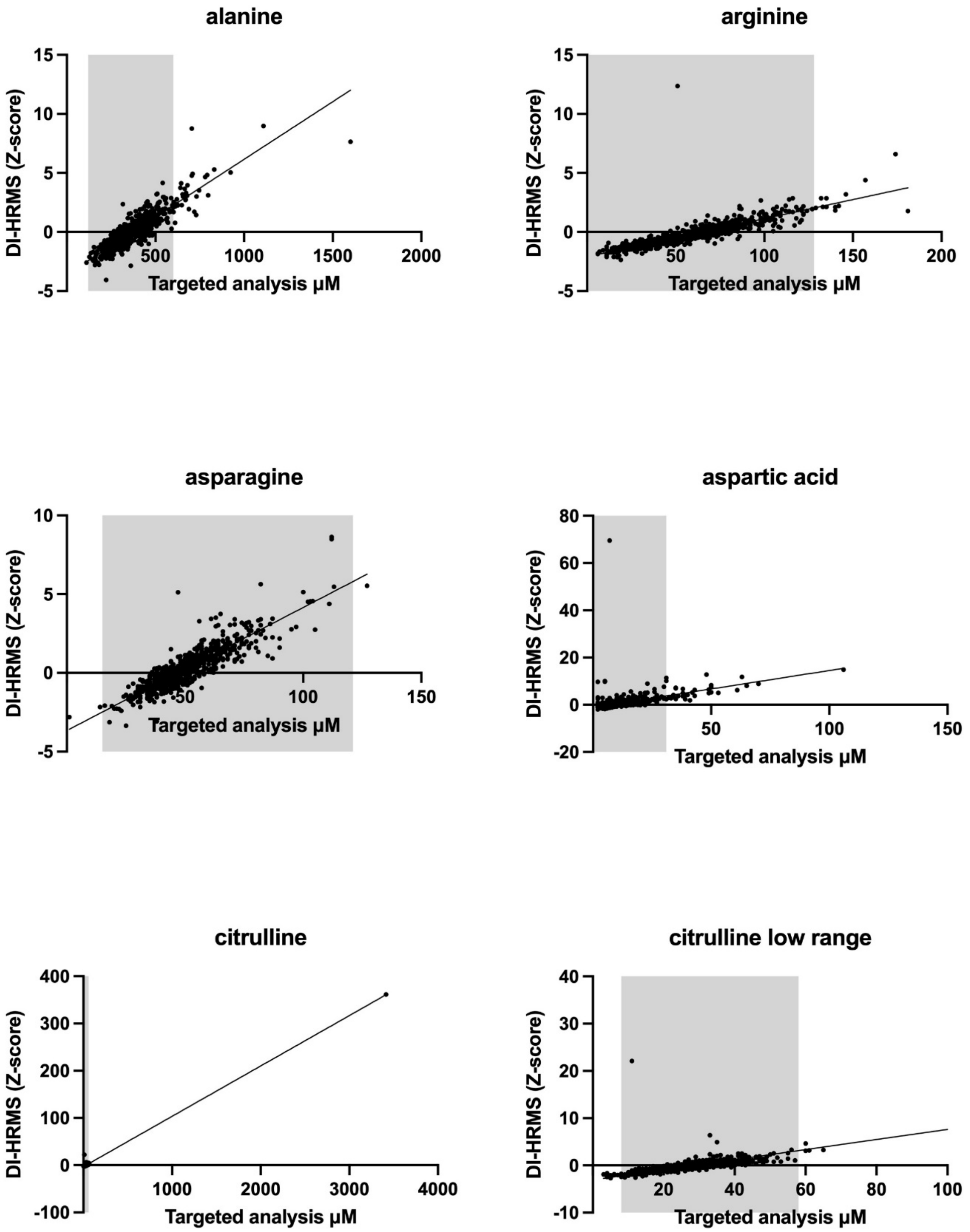
alanine
DI-HRMS (Z-score)
Targeted analysis µM
15
10
5
0
-5
500
1000
1500
2000
arginine
DI-HRMS (Z-score)
Targeted analysis µM
15
10
5
0
-5
50
100
150
200
asparagine
DI-HRMS (Z-score)
Targeted analysis µM
10
5
0
-5
50
100
150
aspartic acid
DI-HRMS (Z-score)
Targeted analysis µM
80
60
40
20
0
-20
50
100
150
citrulline
DI-HRMS (Z-score)
Targeted analysis µM
400
300
200
100
0
-100
1000
2000
3000
4000
citrulline low range
DI-HRMS (Z-score)
Targeted analysis µM
40
30
20
10
0
-10
20
40
60
80
100

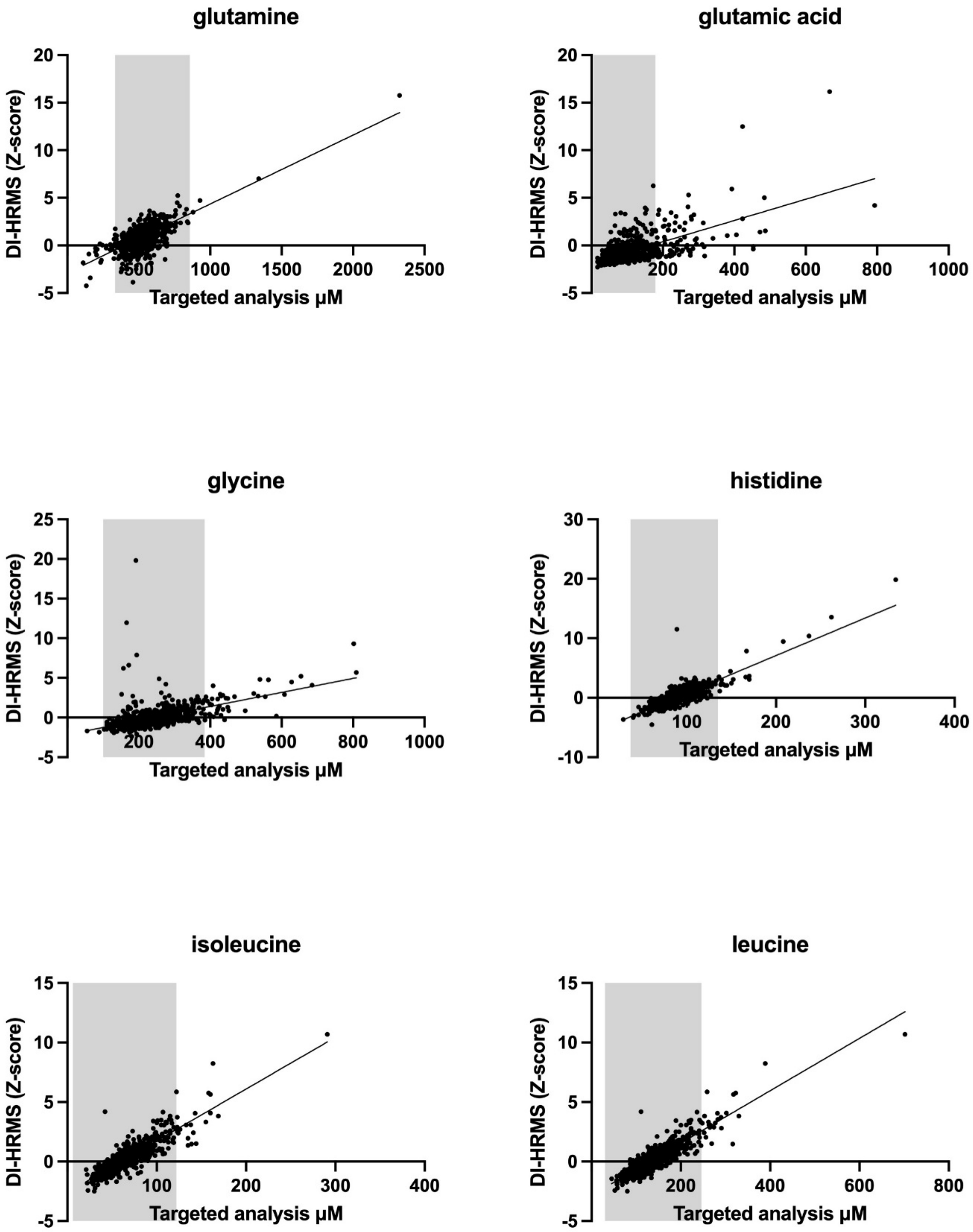
glutamine
DI-HRMS (Z-score)
Targeted analysis µM
glutamic acid
DI-HRMS (Z-score)
Targeted analysis µM
glycine
DI-HRMS (Z-score)
Targeted analysis µM
histidine
DI-HRMS (Z-score)
Targeted analysis µM
isoleucine
DI-HRMS (Z-score)
Targeted analysis µM
leucine
DI-HRMS (Z-score)
Targeted analysis µM

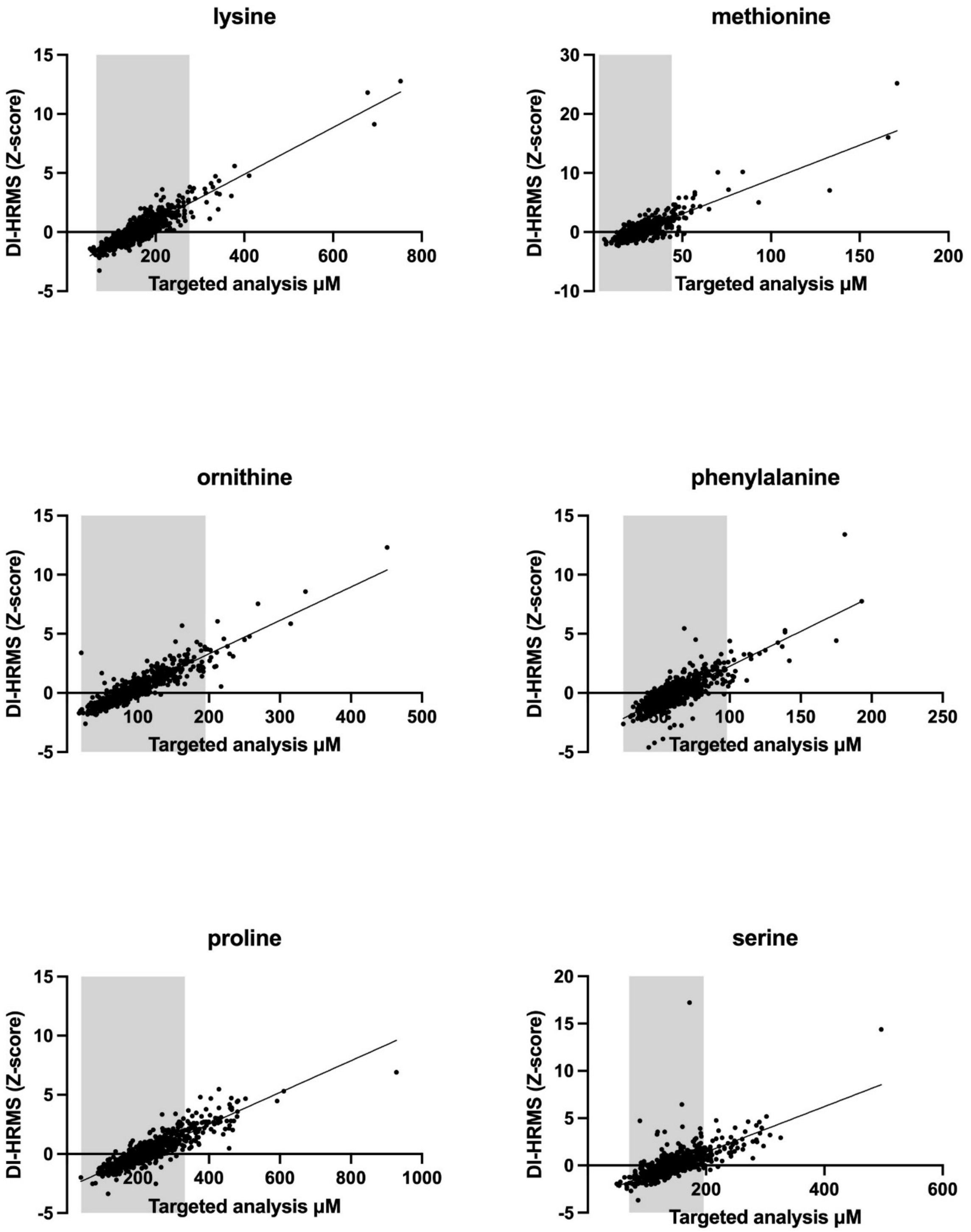
lysine
methionine
ornithine
phenylalanine
proline
serine
DI-HRMS (Z-score)
Targeted analysis µM

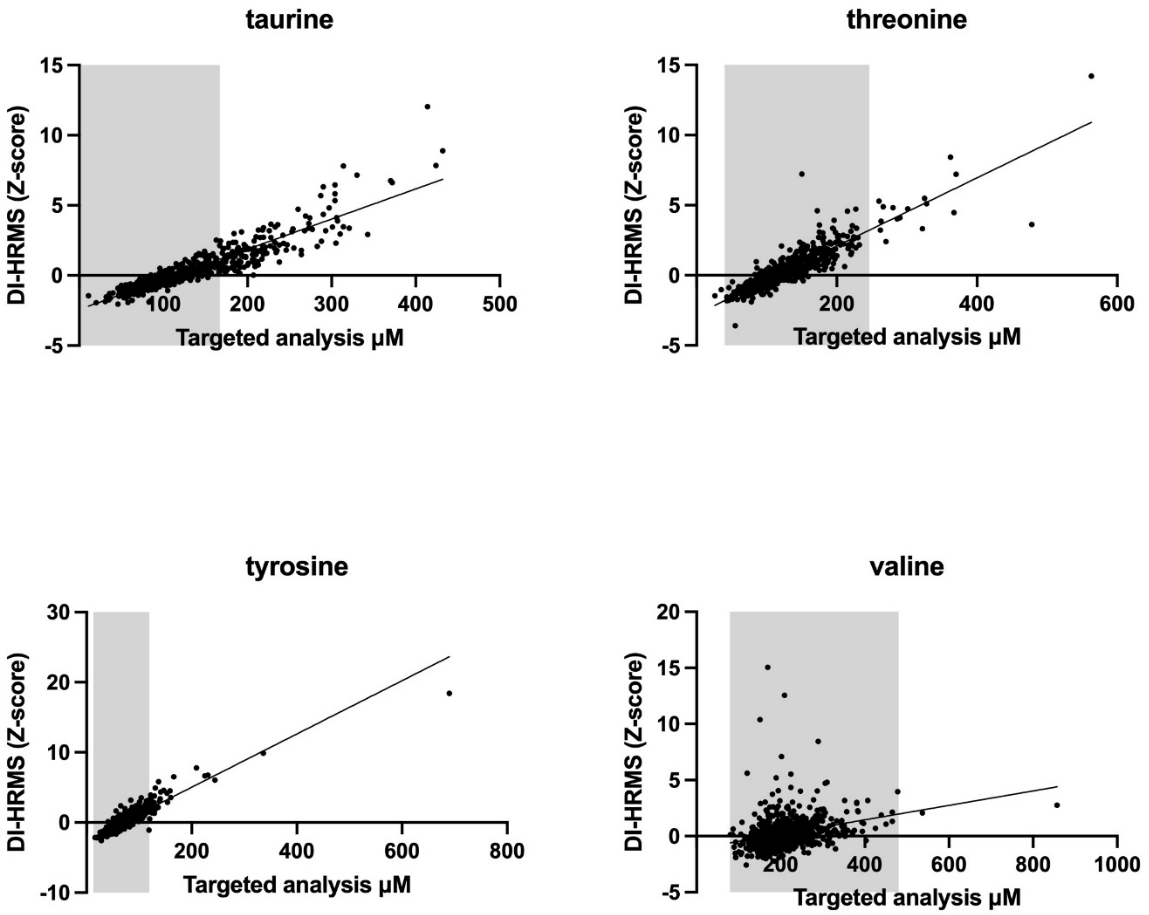

Supplemental figure SF3. Amino acid correlation plots of quantitative concentration analyzed by targeted assays versus semi-quantitative Z-score analyzed by DI-HRMS. Grey areas represent refence ranges. Combined Z-scores for leucine and isoleucine are plotted against individual concentrations because isomeric compounds cannot be separated by DI-HRMS.

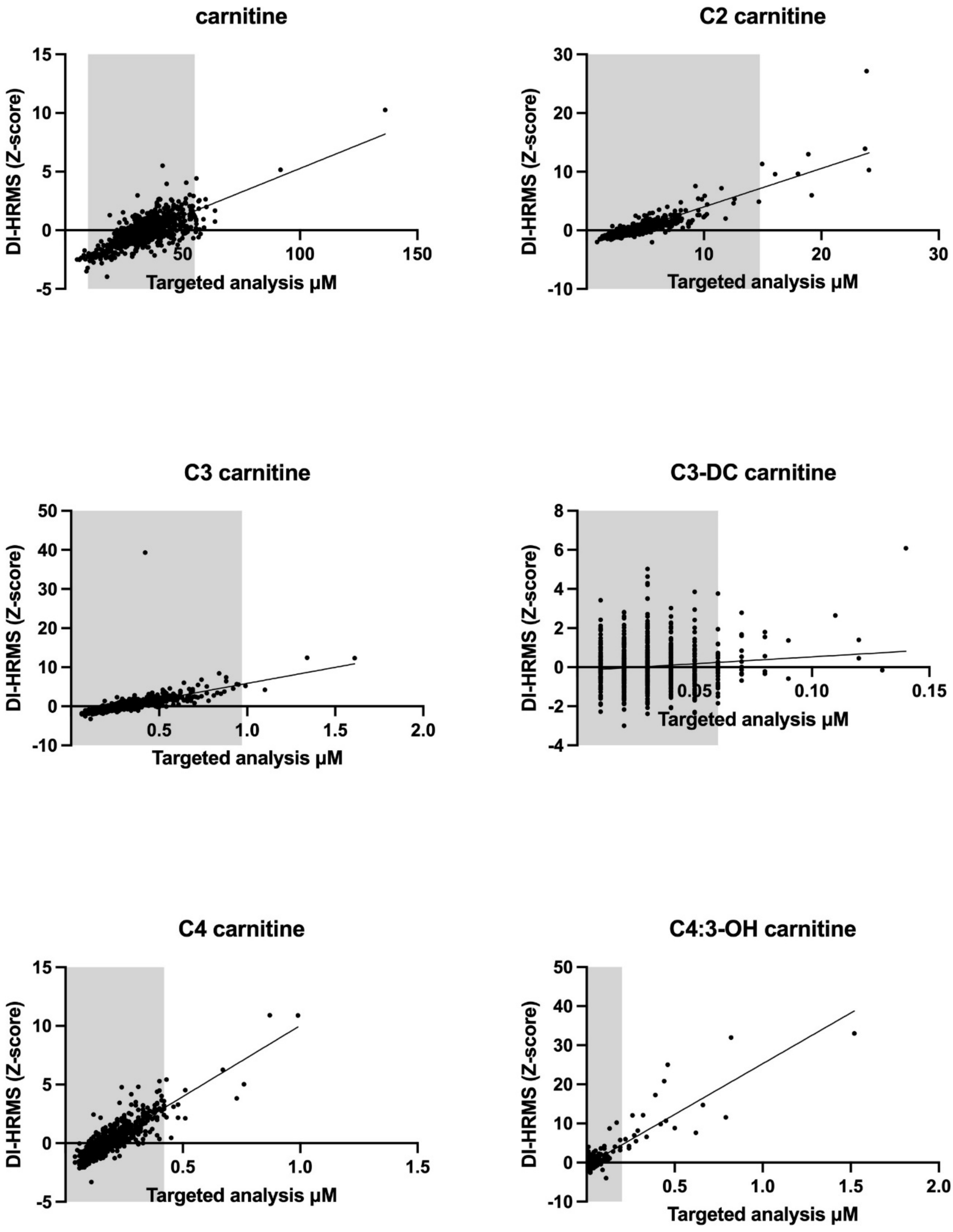
carnitine
DI-HRMS (Z-score)
Targeted analysis µM
15
10
5
0
-5
50
100
150
C2 carnitine
DI-HRMS (Z-score)
Targeted analysis µM
30
20
10
0
-10
10
20
30
C3 carnitine
DI-HRMS (Z-score)
Targeted analysis µM
50
40
30
20
10
0
-10
0.5
1.0
1.5
2.0
C3-DC carnitine
DI-HRMS (Z-score)
Targeted analysis µM
8
6
4
2
0
-2
-4
0.05
0.10
0.15
C4 carnitine
DI-HRMS (Z-score)
Targeted analysis µM
15
10
5
0
-5
0.5
1.0
1.5
C4:3-OH carnitine
DI-HRMS (Z-score)
Targeted analysis µM
50
40
30
20
10
0
-10
0.5
1.0
1.5
2.0

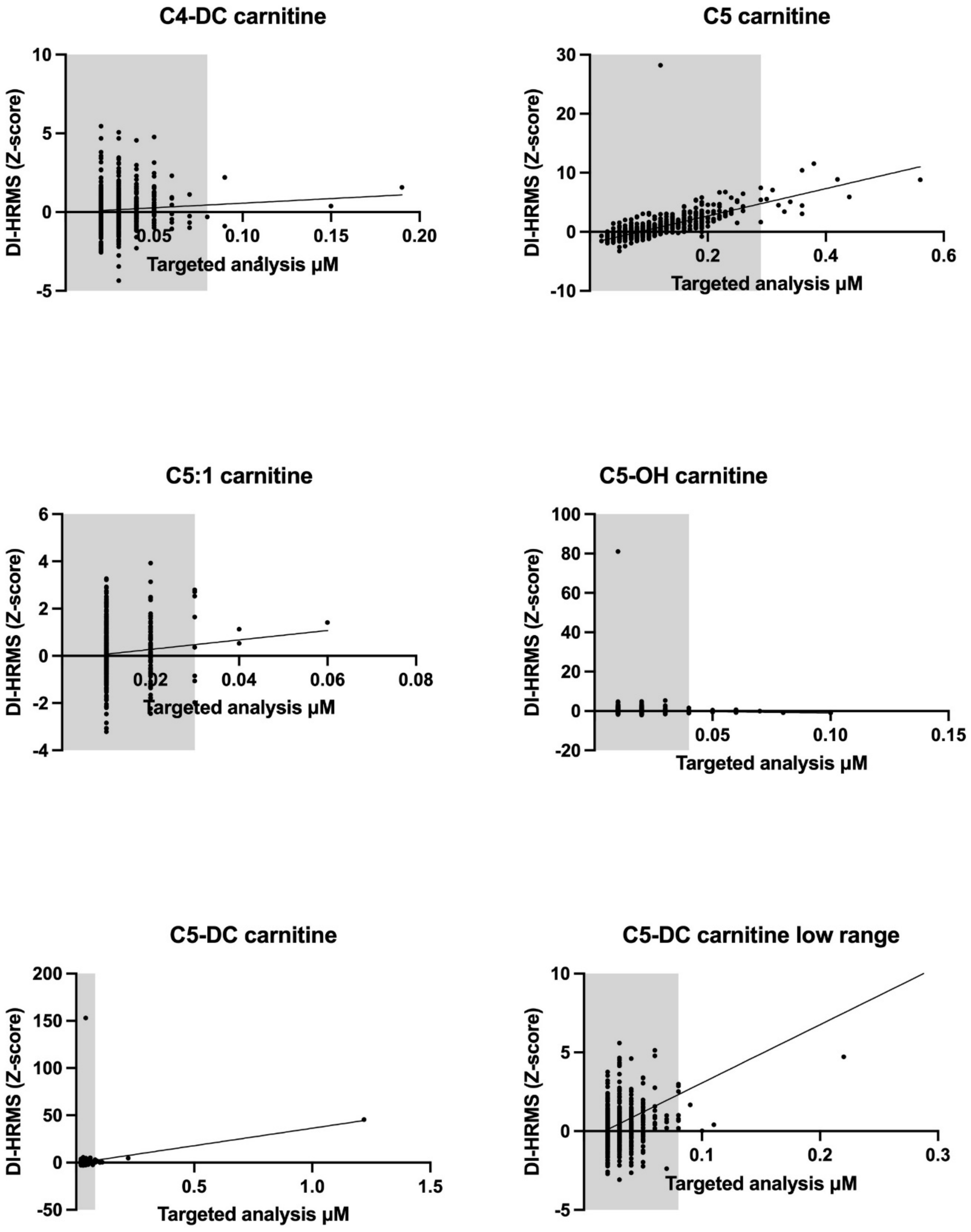
C4-DC carnitine
C5 carnitine
C5:1 carnitine
C5-OH carnitine
C5-DC carnitine
C5-DC carnitine low range
DI-HRMS (Z-score)
Targeted analysis µM

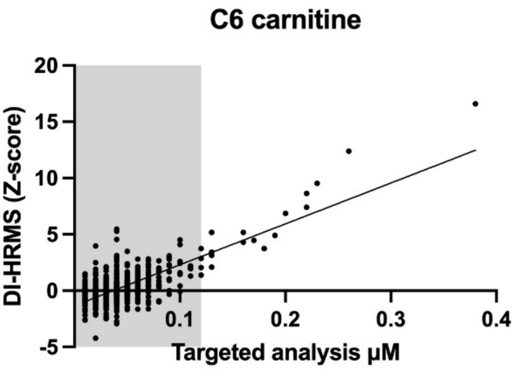
C6 carnitine
DI-HRMS (Z-score)
Targeted analysis µM
20
15
10
5
0
-5
0.1
0.2
0.3
0.4

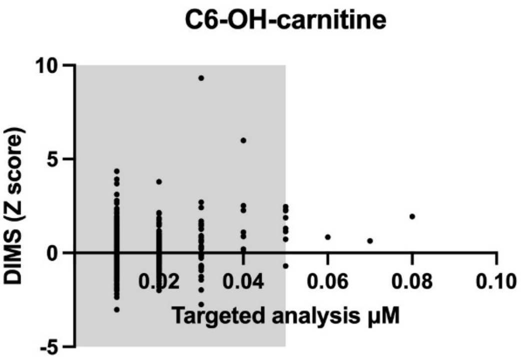
C6-OH-carnitine
DIMS (Z score)
Targeted analysis µM
10
5
0
-5
0.02
0.04
0.06
0.08
0.10

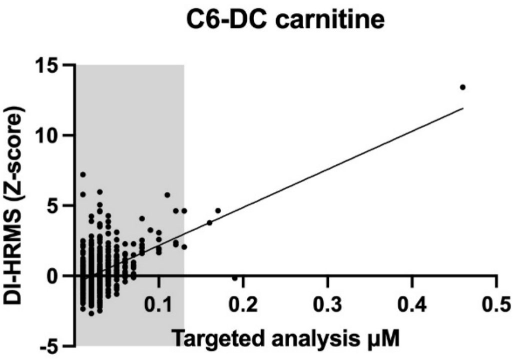
C6-DC carnitine
DI-HRMS (Z-score)
Targeted analysis µM
15
10
5
0
-5
0.1
0.2
0.3
0.4
0.5

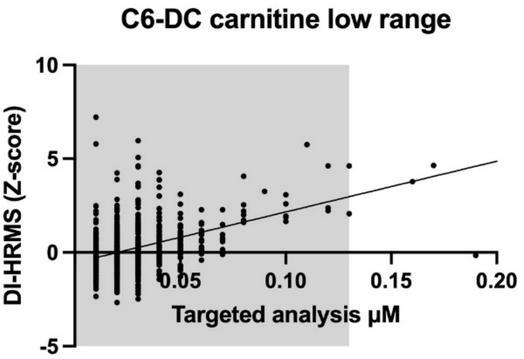
C6-DC carnitine low range
DI-HRMS (Z-score)
Targeted analysis µM
10
5
0
-5
0.05
0.10
0.15
0.20

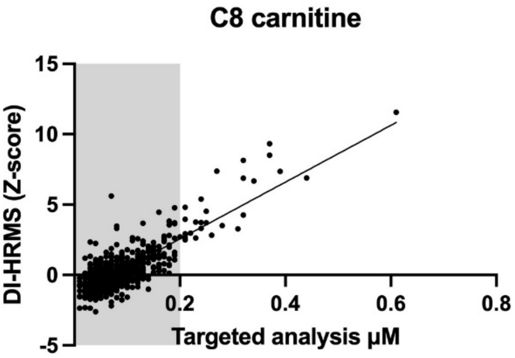
C8 carnitine
DI-HRMS (Z-score)
Targeted analysis µM
15
10
5
0
-5
0.2
0.4
0.6
0.8

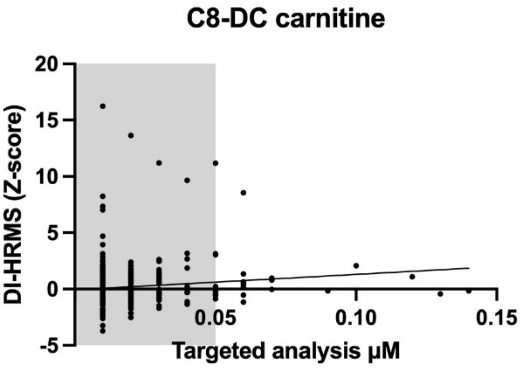
C8-DC carnitine
DI-HRMS (Z-score)
Targeted analysis µM
20
15
10
5
0
-5
0.05
0.10
0.15

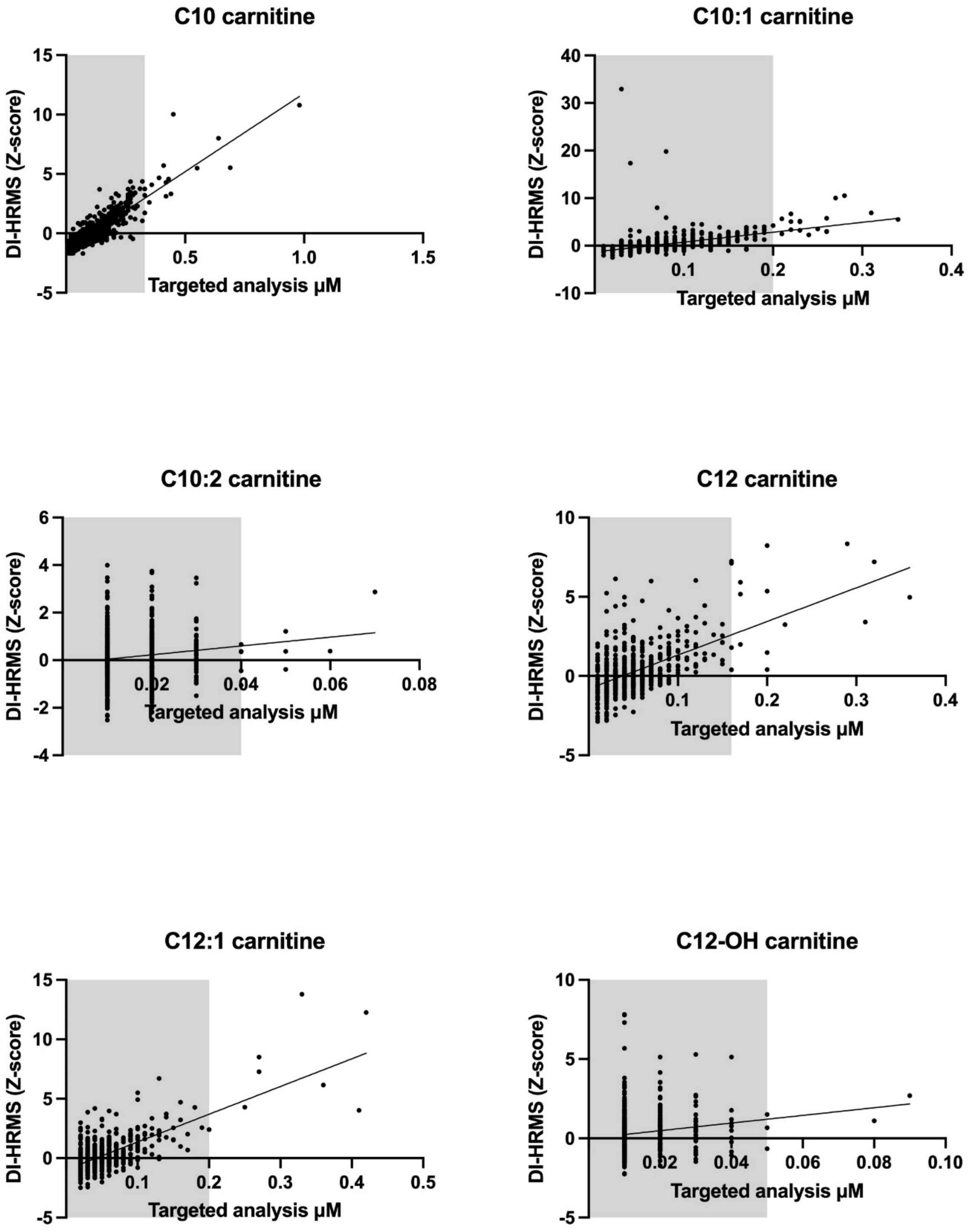
C10 carnitine
DI-HRMS (Z-score)
Targeted analysis µM
15
10
5
0
-5
0.5
1.0
1.5
C10:1 carnitine
DI-HRMS (Z-score)
Targeted analysis µM
40
30
20
10
0
-10
0.1
0.2
0.3
0.4
C10:2 carnitine
DI-HRMS (Z-score)
Targeted analysis µM
6
4
2
0
-2
-4
0.02
0.04
0.06
0.08
C12 carnitine
DI-HRMS (Z-score)
Targeted analysis µM
10
5
0
-5
0.1
0.2
0.3
0.4
C12:1 carnitine
DI-HRMS (Z-score)
Targeted analysis µM
15
10
5
0
-5
0.1
0.2
0.3
0.4
0.5
C12-OH carnitine
DI-HRMS (Z-score)
Targeted analysis µM
10
5
0
-5
0.02
0.04
0.06
0.08
0.10

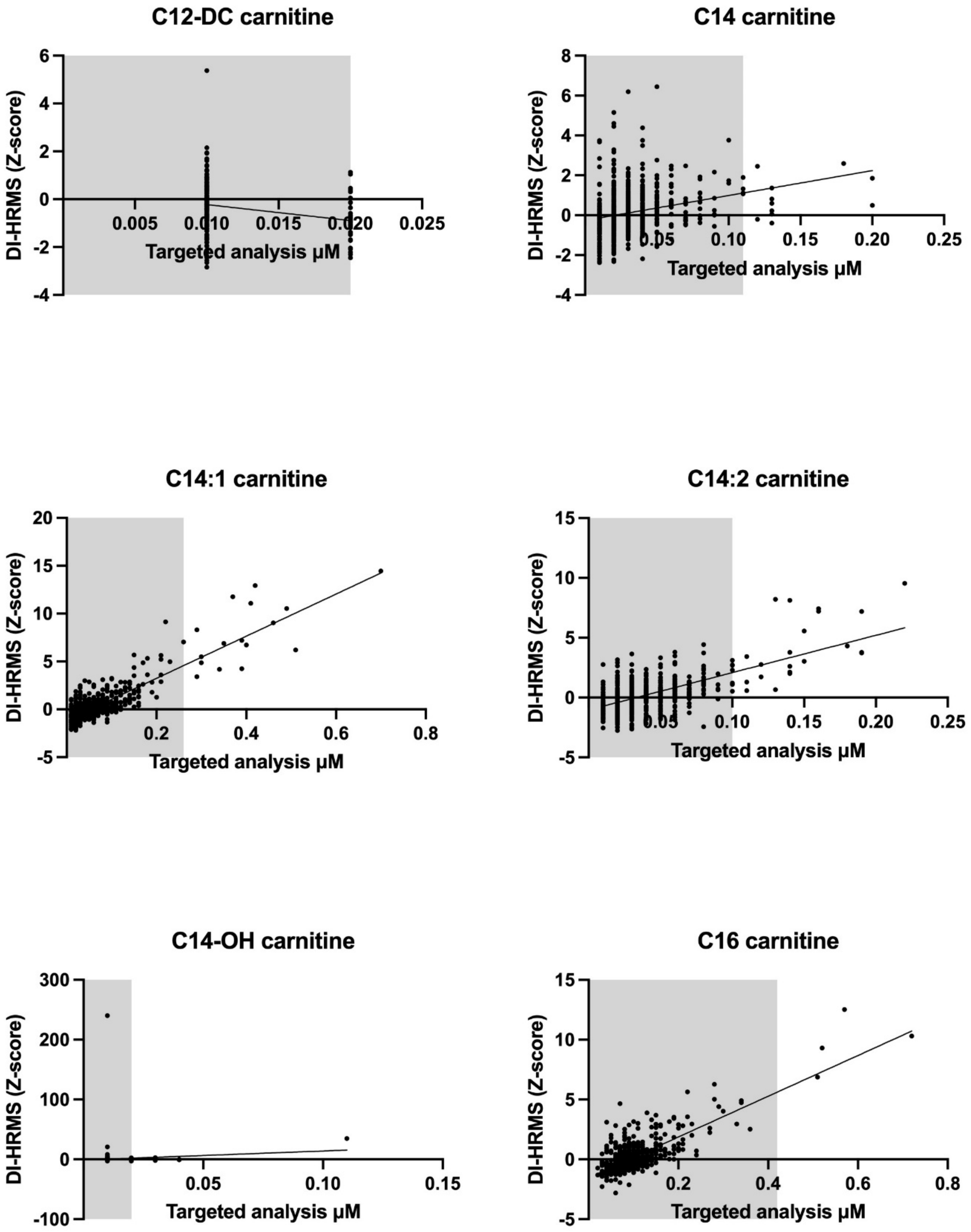

C12-DC carnitine
DI-HRMS (Z-score)
6
4
2
0
-2
-4
0.005
0.010
0.015
0.020
0.025
Targeted analysis μM
C14 carnitine
DI-HRMS (Z-score)
8
6
4
2
0
-2
-4
0.05
0.10
0.15
0.20
0.25
Targeted analysis μM
C14:1 carnitine
DI-HRMS (Z-score)
20
15
10
5
0
-5
0.2
0.4
0.6
0.8
Targeted analysis μM
C14:2 carnitine
DI-HRMS (Z-score)
15
10
5
0
-5
0.05
0.10
0.15
0.20
0.25
Targeted analysis μM
C14-OH carnitine
DI-HRMS (Z-score)
300
200
100
0
-100
0.05
0.10
0.15
Targeted analysis μM
C16 carnitine
DI-HRMS (Z-score)
15
10
5
0
-5
0.2
0.4
0.6
0.8
Targeted analysis μM

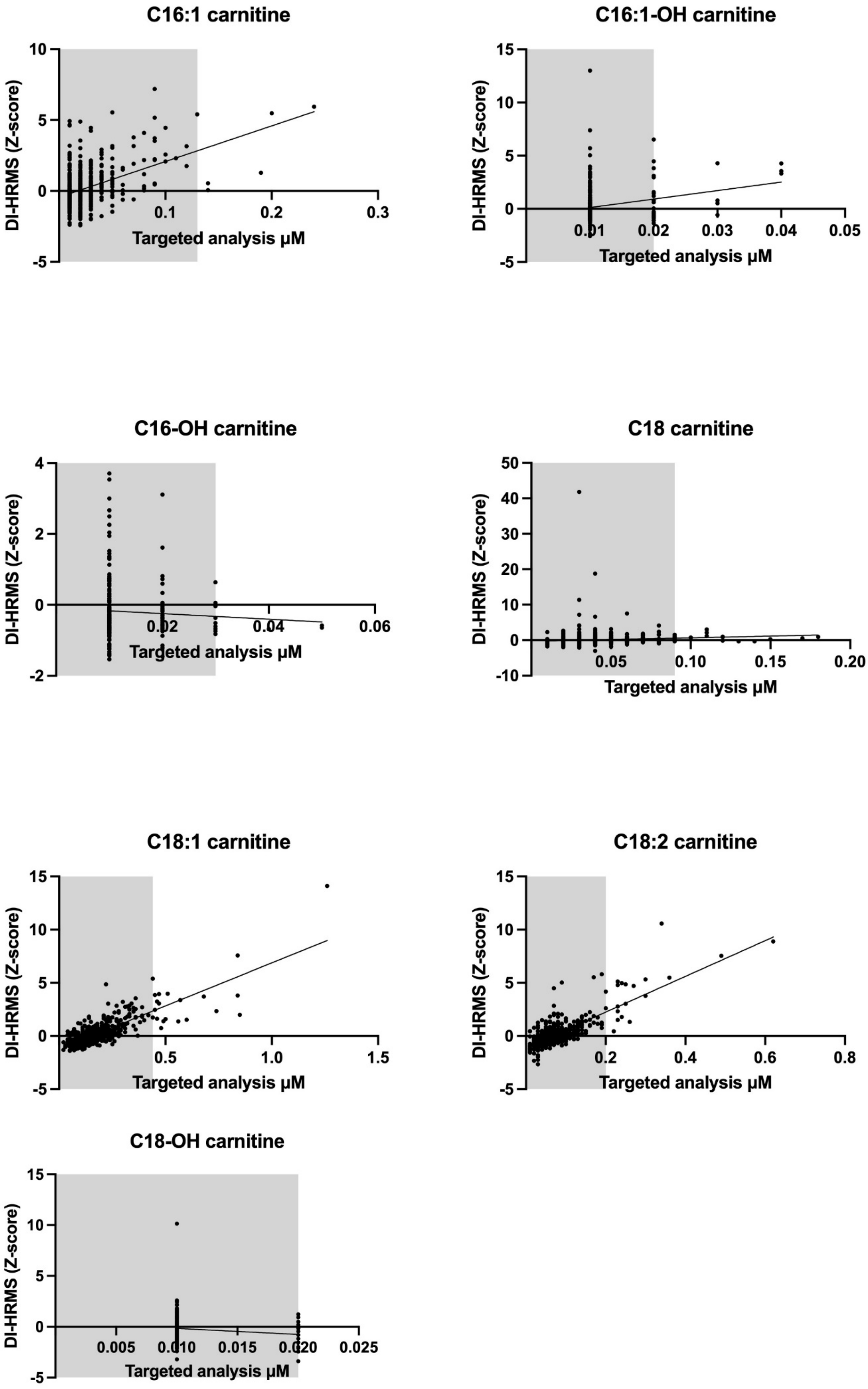
C16:1 carnitine
DI-HRMS (Z-score)
Targeted analysis μM
C16:1-OH carnitine
DI-HRMS (Z-score)
Targeted analysis μM
C16-OH carnitine
DI-HRMS (Z-score)
Targeted analysis μM
C18 carnitine
DI-HRMS (Z-score)
Targeted analysis μM
C18:1 carnitine
DI-HRMS (Z-score)
Targeted analysis μM
C18:2 carnitine
DI-HRMS (Z-score)
Targeted analysis μM
C18-OH carnitine
DI-HRMS (Z-score)
Targeted analysis μM

Supplemental figure SF4. Acylcarnitine correlation plots of quantitative concentration analyzed by targeted assays versus semi-quantitative Z-score analyzed by DI-HRMS.

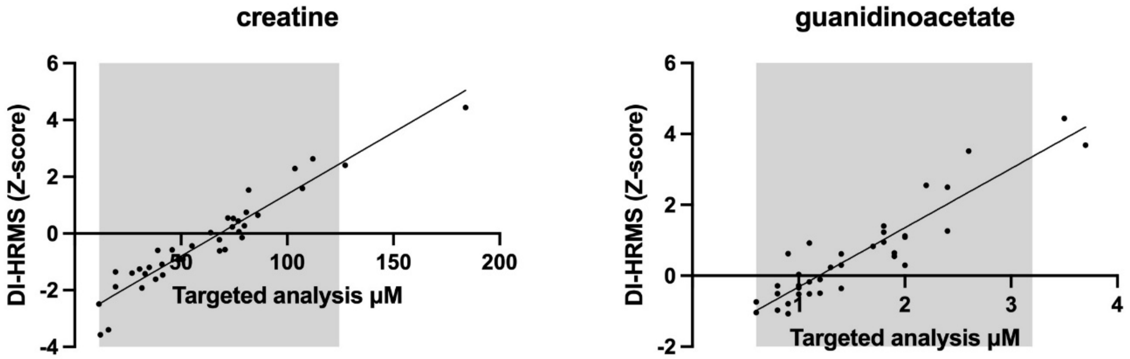

Supplemental figure SF5. Creatine and guanidinoacetate correlation plots of quantitative concentration analyzed by targeted assays versus semi-quantitative Z-score analyzed by DI-HRMS.

	Pearson's correlation coefficient	p	N (X,Y pairs)
Alanine	0,8788	<0,0001	710
Arginine	0,8244	<0,0001	710
Asparagine	0,8772	<0,0001	709
Aspartic acid	0,4812	<0,0001	710
Citrulline	0,9969	<0,0001	710
Glutamine	0,7056	<0,0001	710
Glutamic acid	0,6086	<0,0001	710
Glycine	0,5036	<0,0001	710
Histidine	0,8452	<0,0001	710
Isoleucine	0,8691	<0,0001	710
Leucine	0,8901	<0,0001	710
Lysine	0,9189	<0,0001	710
Methionine	0,8478	<0,0001	710
Ornithine	0,9111	<0,0001	710
Phenylalanine	0,8243	<0,0001	710
Proline	0,8978	<0,0001	710
Serine	0,7072	<0,0001	710
Taurine	0,9237	<0,0001	710
Threonine	0,8907	<0,0001	710
Tyrosine	0,9168	<0,0001	710
Valine	0,2951	<0,0001	710

Supplemental table ST1. Amino acid correlation coefficients of quantitative concentration analyzed using targeted assays versus semi-quantitative Z-score obtained by DI-HRMS. Combined Z-scores for leucine and isoleucine are used against individual concentrations because isomeric compounds cannot be separated by DI-HRMS.

	Pearson's correlation coefficient	p	N (X,Y pairs)
Carnitine	0,7396	<0,0001	704
C2-carnitine	0,8834	<0,0001	704
C3-carnitine	0,6538	<0,0001	704
C3-DC-carnitine	0,1048	0,0054	703
C4-carnitine	0,8634	<0,0001	704
C4:3-OH-carnitine	0,8825	<0,0001	635
C4-DC-carnitine	0,06882	0,0766	663
C5-carnitine	0,7154	<0,0001	704
C5:1-carnitine	0,09659	0,0373	465
C5-OH-carnitine	-0,03835	0,3362	631
C5-DC-carnitine	0,2844	<0,0001	639
C6-carnitine	0,7488	<0,0001	701
C6-DC-carnitine	0,5159	<0,0001	690
C8-carnitine	0,8093	<0,0001	702
C8-DC-carnitine	0,1205	0,0146	410
C10-carnitine	0,8737	<0,0001	703
C10:1-carnitine	0,4556	<0,0001	703
C10:2-carnitine	0,1316	<0,0011	613
C12-carnitine	0,5684	<0,0001	696
C12:1-carnitine	0,6915	<0,0001	610
C12-OH-carnitine	0,1618	0,0003	488
C12-DC-carnitine	-0,2315	0,0008	206
C14-carnitine	0,2557	<0,0001	692
C14:1-carnitine	0,8531	<0,0001	697
C14:2-carnitine	0,6241	<0,0001	694
C14-OH-carnitine	0,08369	0,1155	355
C16-carnitine	0,7629	<0,0001	704
C16:1-carnitine	0,4530	<0,0001	654
C16:1-OH-carnitine	0,2307	<0,0001	317
C16-OH-carnitine	-0,05814	0,3770	233
C18-carnitine	0,09670	0,0104	702
C18:1-carnitine	0,8081	<0,0001	704
C18:2-carnitine	0,7751	<0,0001	704
C18-OH-carnitine	-0,1264	0,1004	170

Supplemental table ST2. Acylcarnitine correlation coefficients of quantitative concentration analyzed using targeted assays versus semi-quantitative Z-score obtained by DI-HRMS.

	Pearson's correlation coefficient	p	N (X,Y pairs)
Creatine	0,9547	<0,0001	38
Guanidinoacetate	0,9203	<0,0001	37

Supplemental table ST3. Creatine and guanidinoacetate correlation coefficients of quantitative concentration analyzed using targeted assays versus semi-quantitative Z-score obtained by DI-HRMS.

Sample	IMD (group)	Aberration	Found with DI-HRMS?
id427	Amino acid metabolism	THR,ASP,GLU,ALA,CIT,LEU,(ALLO)ILE,TYR,ORN,LYS,HIS↓	Yes
id266	Amino acid metabolism; Fatty acid oxidation defect	PRO,C6,C8,C10,C12,C14:1,C14,C16:1,C14:1/C2,(C16+C18:1)/ C2↑ CIT,VAL,LEU,ARG,C0 ↓	Yes;Yes
id190	Argininosuccinate synthetase deficiency (ASS1)	CIT,GLN ↑ ORN ↓	Yes
id738	Asparagine synthetase deficiency (ASNS)	ASN↓	Yes
id595	Carnitine palmitoyltransferase 2 deficiency (CPT2)	C18:1,C16+C18:1/C2↑	No
id165	Fatty acid oxidation defect	C6,C8,C10:1,C10,C8/C10↑	Yes
id167	Fatty acid oxidation defect	C4:3-OH,C12,C14:1,C14:2,C14↑	Yes
id215	Fatty acid oxidation defect	C10:1;C12;C14:2,C14:1,C14:1/C2↑	Yes
id146	Fatty acid oxidation defect; Proline metabolism or mitochondrial dysfunction	C3,C4,C6,C8,C8-DC,C8/C10,PRO↑	Yes;Yes
id729	Glutaryl-CoA dehydrogenase deficiency (GCDH)	C0↓,C5-DC↑	Yes
id339	Ketone body metabolism defect	C4:3-OH↑	Yes
id198	Medium-chain acyl CoA dehydrogenase deficiency (ACADM)	C0↓ C6,C8,C8/C10↑	Yes
id548	Medium-chain acyl CoA dehydrogenase deficiency (ACADM)	C8,C10:1,C8/C10↑	Yes
id014	Mitochondrial dysfunction	PRO,ALA↑	Yes
id048	Mitochondrial dysfunction	PRO,ALA↑	Yes
id056	Mitochondrial dysfunction	PRO,ALA↑	Yes
id096	Mitochondrial dysfunction	CIT↓	No
id138	Mitochondrial dysfunction	ALA↑	Yes
id184	Mitochondrial dysfunction	PRO,GLY,ALA,LYS↑	Yes
id192	Mitochondrial dysfunction	ALA↑	Yes
id223	Mitochondrial dysfunction	ALA↑	Yes

id225	Mitochondrial dysfunction	PRO,ALA↑	Yes
id263	Mitochondrial dysfunction	PRO,ALA↑	Yes
id271	Mitochondrial dysfunction	PRO,GLY,ALA↑	Yes
id300	Mitochondrial dysfunction	PRO,ALA↑	Yes
id312	Mitochondrial dysfunction	PRO,ALA↑	No
id414	Mitochondrial dysfunction	ALA↑	Yes
id446	Mitochondrial dysfunction	PRO,ALA↑	No
id453	Mitochondrial dysfunction	ALA↑	No
id476	Mitochondrial dysfunction	PRO↑	Yes
id646	Mitochondrial dysfunction	PRO,ALA↑	Yes
id663	Mitochondrial dysfunction	PRO,ALA↑	Yes
id669	Mitochondrial dysfunction	ALA↑	Yes
id682	Mitochondrial dysfunction	PRO,ALA↑	Yes
id677	Mitochondrial dysfunction	PRO,ALA↑	Yes
id561	Mitochondrial dysfunction	PRO,ALA↑	Yes
id564	Mitochondrial dysfunction	PRO,ALA↑	Yes
id685	Mitochondrial dysfunction	ALA↑	No
id790	Mitochondrial dysfunction	PRO,GLY,ALA↑	Yes
id550	Mitochondrial dysfunction	PRO,ALA↑	Yes
id576	Mitochondrial dysfunction; Carnitine palmitoyltransferase 2 deficiency (CPT2)	PRO,ALA,C18:1,C18:2↑	Yes;Yes
id569	Mitochondrial dysfunction; Fatty acid oxidation defect	PRO,GLY,ALA;C3;C4;C5;C6;C8;C10;C14:2↑	Yes;Yes
id702	Proline metabolism or mitochondrial dysfunction	PRO↑	Yes
id749	Proline metabolism or mitochondrial dysfunction	PRO↑	Yes
id066	Proline metabolism or mitochondrial dysfunction	PRO,GLY↑	Yes
id089	Proline metabolism or mitochondrial dysfunction	PRO↑	Yes

id100	Proline metabolism or mitochondrial dysfunction	PRO↑	Yes
id117	Proline metabolism or mitochondrial dysfunction	PRO↑	Yes
id120	Proline metabolism or mitochondrial dysfunction	PRO↑	Yes
id183	Proline metabolism or mitochondrial dysfunction	PRO↑	Yes
id261	Proline metabolism or mitochondrial dysfunction	PRO↑	Yes
id274	Proline metabolism or mitochondrial dysfunction	PRO↑	No
id283	Proline metabolism or mitochondrial dysfunction	PRO↑	No
id297	Proline metabolism or mitochondrial dysfunction	PRO↑	No
id387	Proline metabolism or mitochondrial dysfunction	PRO↑	Yes
id399	Proline metabolism or mitochondrial dysfunction	PRO↑	Yes
id520	Proline metabolism or mitochondrial dysfunction	PRO↑	Yes
id547	Proline metabolism or mitochondrial dysfunction	PRO↑	Yes
id249	Riboflavine deficiency or defect	C6,C8,C10↑	Yes
id176	Riboflavine deficiency or defect	C4,C5,C6,C8,C8/C10↑	Yes
id474	Riboflavine deficiency or defect	C4:3-OH,C8,C10,C6,C14:1↑	Yes
id643	Riboflavine deficiency or defect	C8,C10:1,C10,C3-DC,C12:1,C12,C14:1↑	Yes
id277	Tyrosinemia	TYR↑	Yes
id311	Very long-chain acyl CoA dehydrogenase deficiency (ACADVL)	C14:2,C14:1↑	Yes

Supplemental table ST5. Overview of samples with aberrant metabolites possibly indicative of IMD, based on targeted assays. The fourth column shows whether this aberration was also detected with DI-HRMS.
